# Annotations

**Published:** 1954-10

**Authors:** 


### Annotations

### EXTENSIONS TO THE UNIVERSITY DEPARTMENT OF MEDICINE

During the winter there were important extensions to the University Department of Medicine. The cramped attic-rooms at the top of the Infirmary (which had been used for occupational therapy) were demolished and replaced by a well-equipped lecture theatre. This theatre was sorely needed. It has been designed not only for lectures but for clinical demonstrations and film projections. It adds much to the amenities for undergraduate and post-graduate education.

There are several other new rooms suitable for use either as separate laboratories or private offices.

### TOO MANY MEDICO-SOCIAL WORKERS?

We are all familiar with the increasing number of specialties within the ranks of the medical profession; indeed, it is becoming quite difficult to keep track of the many different " -ologists " and " -icians " who minister to our present-day needs. What may not be quite so well known is that a parallel development has been going on in the medico-social field.

Dr. R. C. Wofinden, in a recent article,[1] published in *The Medical Officer*, draws our attention to the wide variety of medico-social workers. He found that there were no less than thirty-five different medico-social workers, visiting the homes of Bristol people.

The author conducted a pilot survey on approximately a thousand families who were being visited in their homes by health visitors. The sample was heavily biased by families with mothers between the ages 20–45 and therefore firm conclusions may not be drawn from the findings. Nearly 14 per cent. of the families had been visited by three or more different workers in addition to the health visitor. Most of these families were of the " problem family " type, where multiplicity of visiting is a well-known phenomenon. Multiple visiting was more frequent in families

[1] *Med. Offr.* **91**, 83–5. ' A note on multiplicity of home visiting by medico-social workers ', R. C. Wofinden, M.D., D.P.H., Deputy Medical Officer of Health, Bristol.

in which there were more children in the household, greater degrees of overcrowding and poor social and evironmental standards, so the author concludes that most effort appears to be made on behalf of those most likely to be in need of assistance.

After reading this article we look forward, with interest, to the findings and recommendations of the Ministerial Working Parties, which are at present studying the function of the Health Visitor, the Home Nurse and other social workers.

### *News from Societies*

